# Recombinant *Leishmania*-activated C kinase as a novel antigenic candidate for immuno-diagnosis of visceral leishmaniasis occurring in India and Brazil

**DOI:** 10.1186/s40249-025-01296-7

**Published:** 2025-07-09

**Authors:** Anirban Bhattacharyya, Nicky Didwania, Sarfaraz Ahmad Ejazi, Rudra Chhajer, Saswati Gayen, Mehebubar Rahman, Rama Prosad Goswami, Krishna Pandey, Vidya Nand Ravi Das, Pradeep Das, Fernando Oliveira da Silva, Dorcas Lamounier Costa, Carlos Henrique Nery Costa, Nahid Ali

**Affiliations:** 1 (CSIR)-Indian Institute of Chemical Biology, Infectious Diseases and Immunology Division, 4, Raja S.C. Mullick Road, Jadavpur, Kolkata, West Bengal India; 2 Department of Tropical Medicine, School of Tropical Medicine, Kolkata, West Bengal India; 3 Department of Molecular Biology, Rajendra Memorial Research Institute of Medical Sciences, Patna, Bihar India; 4 Department of Microbiology, Vijaygarh Jyotish Ray College, 8/2, Bejoygarh, Jadavpur, Kolkata, West Bengal India; 5 Present Address: Fischell Department of Bioengineering, A. James Clark School of Engineering, University of Maryland, College Park, MD USA; 6 The Federal University of Piauí (UFPI), Teresina, Piauí Brazil

**Keywords:** *L. donovani* activated C kinase (LACK), Visceral leishmaniasis, Diagnosis, ELISA, Dipstick

## Abstract

**Background:**

Visceral leishmaniasis (VL) an ‘infectious disease of poverty’, caused by the *Leishmania donovani* complex, remains a significant public health threat in endemic regions of South Asia, East Africa, and Brazil. Early and accurate diagnosis is critical to prevent the disease's potentially fatal outcomes. However, due to the nonspecific nature of clinical symptoms, diagnosis often relies on serological tests. This study aims to assess the diagnostic potential of the *L. donovani* activated C kinase (LACK), a highly conserved antigen essential for parasite survival and host establishment, in VL-endemic regions such as India and Brazil.

**Methods:**

We conducted a multi-center study with serum samples from India (*n* = 184) and Brazil (*n* = 59), along with non-invasive urine samples from India (*n* = 132). Clinical samples from India were collected from the endemic regions of Bihar and West Bengal between 2016–2024, while those from Teresina, Brazil, were collected between 2008 and 2009. Following preliminary immunoblot analysis, we validated the diagnostic utility of LACK through enzyme-linked immunosorbent assays (ELISA) and dipstick tests. Results were analyzed and area under a Receiver Operating Characteristic (ROC) curve (AUC) values were calculated via the Mann–Whitney U test. Additionally, sensitivity, specificity, and confidence intervals were assessed to evaluate diagnostic performance.

**Results:**

The ELISA results revealed that LACK antibodies exhibited 100% sensitivity in both Indian [95% confidence intervals (*CI*): 94.80–100%] and Brazilian (95% *CI*: 91.24–100%) patient samples, with specificity of 97.33% for Indian controls and 94.74% for Brazilian controls. Urine samples from Indian patients also demonstrated perfect sensitivity and specificity (100%). Notably, LACK showed minimal reactivity with follow-up patient samples. Dipstick assays confirmed these findings, offering a simple, rapid, and field-friendly diagnostic alternative.

**Conclusion:**

LACK is a promising diagnostic marker for VL, showing high sensitivity across regions and has potential to distinguish active infections from cured or relapsed cases, though larger studies are needed for confirmation.

**Graphical Abstract:**

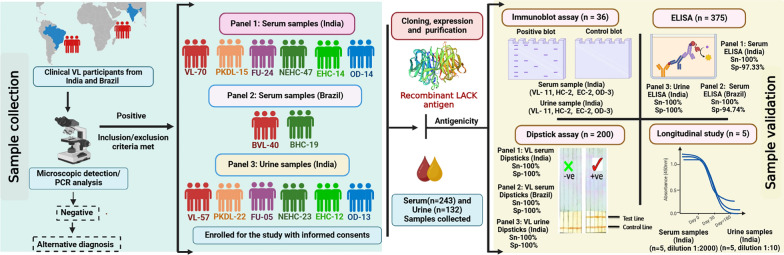

## Background

Visceral leishmaniasis (VL) or kala-azar is a significant public health concern in South Asia, East Africa, and Brazil, where recurrent outbreaks result in severe clinical manifestations and high rates of relapse and mortality. Factors such as poverty, malnutrition, and population movements in endemic regions exacerbate the risk of developing VL [1]. In India, the disease is primarily caused by *Leishmania donovani*, while in Brazil, it is mainly associated with *Leishmania infantum*, both transmitted by the bites of infected female sandflies, which require human blood to reproduce [2]. According to the WHO's 2022 report, VL is endemic in 80 countries globally. Notably, Eastern Africa—comprising Chad, Eritrea, Ethiopia, Kenya, Somalia, South Sudan, Sudan, and Uganda—accounts for 73% of global VL cases, with half of these cases occurring in children under 15 years of age. Brazil represents 16% of cases, and the Indian subcontinent (including Bangladesh, India, and Nepal) is nearing the elimination of VL as a public health issue, yet still contributes to 10% of worldwide cases [1]. These regions are recognized as the three primary eco-epidemiological hotspots for VL.

Kala-azar typically presents with symptoms similar to malaria and dengue, such as irregular fever, weight loss, splenomegaly, hepatomegaly, and anemia. A recent report highlighted a case of VL with multiple relapses and atypical symptoms, eventually leading to neurological complications [1, 3]. Post-kala-azar dermal leishmaniasis (PKDL) can occur in up to 15% of cases within one to ten years after apparent cure of kala-azar, particularly in South-East Asia and East Africa, where kala-azar and PKDL are the predominant forms of the disease [4]. Laboratory diagnosis of VL typically relies on the microscopic examination of amastigotes in Giemsa-stained tissue aspirates, which, while being the gold standard, often delays treatment due to lengthy processing times [5]. Recently, advancements include development of a computer-aided system designed to automatically detect the *Leishmania* parasite in Giemsa-stained microscopic images, but challenges remain, particularly the invasive and uncomfortable nature of bone marrow and splenic aspirations, which require specialized expertise and access to hospital facilities that may not be available in field settings [6]. Furthermore, the Infectious Diseases Society of America (IDSA) guidelines recommend against splenic aspiration for the diagnosis of VL in North America due to the associated high risks, including the potential for severe, life-threatening hemorrhages [7].

Other established diagnostic methods for *Leishmania* infection, such as qualitative tests for detecting *Leishmania* antibodies, include western blot (WB) and immunochromatographic tests (ICT) [8]. Quantitative assays, such as direct agglutination tests (DAT), indirect immunofluorescence (IIF), indirect hemagglutination (IHA), and enzyme-linked immunosorbent assays (ELISA), are also commonly used. However, these methods have notable limitations, including lengthy incubation periods and variable performance, which can impact their reliability [9, 10]. Molecular techniques, like quantitative real-time polymerase chain reaction (qPCR) and loop-mediated isothermal amplification (LAMP), offer high specificity and sensitivity but come with the drawback of higher costs due to the need for specialized laboratory equipment and reagents [5, 11]. Rapid diagnostic tests (RDTs), including lateral flow assays (LFA) and immunodot tests, are under development for VL, particularly in endemic regions of the Indian subcontinent and the Americas [12]. However, existing RDTs, such as the rK39 antigen test, have limitations; they are validated only for patients with fever lasting two weeks or more and cannot distinguish between current and past infections, leading to a decreased positive predictive value in near-elimination scenarios [13, 14]. To enhance VL diagnosis, there is a pressing need to identify new diagnostic antigens. Previous studies on *Leishmania* membrane antigens (LAg) derived from *L. donovani* demonstrated strong sensitivity and specificity for detecting infection-specific antibodies in serum and urine samples from patients with active VL [15, 16]. However, issues like batch-to-batch variability in LAg production and increased costs associated with large-scale production necessitate the search for more reliable antigenic biomarkers. Further improved sensitivity and specificity in diagnostic tests can also facilitate more accurate assessment of treatment efficacy [17].

The *Leishmania* kinome comprises 175–195 protein kinases, which are crucial for regulating cellular signaling that supports parasite’s survival, including processes such as cell cycle progression, differentiation, and virulence throughout its life cycle [18]. While recent advances in bioinformatics and biotechnology have identified protein kinases (PKs) as promising therapeutic targets for leishmaniasis, they have not yet been explored as potential diagnostic markers for visceral leishmaniasis (VL) [19]. One key protein in this context is the *Leishmania* homologue of activated C kinase (LACK), a 34 kDa protein that belongs to a family characterized by tryptophan-aspartic acid dipeptide-40 (WD-40) repeat motifs [20]. LACK plays a critical role in cellular processes essential for *Leishmania* infection and is conserved across species that cause visceral leishmaniasis, with low homology to the host orthologue, making it a potential target for both therapeutic and diagnostic applications [21]. Given its significance, earlier research highlighted LACK as a potential candidate for vaccine development [22]. Studies have shown that when the LACK gene is conjugated with plasmids or DNA demonstrated protective effects against *Leishmania major* infection in mice when administered with viral vectors as boosters [23, 24]. Alonso et al. reported that intranasal homologous prime/boost inoculation of a non-replicative, antibiotic-free DNA vaccine encoding the *L. infantum* LACK gene provides high protection, reducing clinical signs and parasite burden by over 90% in vaccinated dogs. Many studies on LACK immunogenicity have been conducted over the past two decades, including second- and third-generation vaccine formats in mice and Beagle dogs [25]. For the first time, we assessed the reactogenicity of the recombinant LACK antigen by testing its interaction with antibodies from VL patients in two endemic countries, India and Brazil. We screened sera from VL patients in both countries, as well as urine samples from Indian VL patients. The aim of this study was to evaluate LACK as a promising diagnostic marker for VL, with the ability to distinguish between active infections and past exposures.

## Methods

### Sample collection

A total of 191 Indian participants were enrolled in this study across three research sites: The School of Tropical Medicine (STM), Kolkata; Rajendra Memorial Research Institute of Medical Sciences (RMRIMS), Patna; and the CSIR-Indian Institute of Chemical Biology (IICB), Kolkata, between 2016 and 2024. Serum and urine samples were collected after obtaining written informed consent from each participant, with consent forms tailored to the local languages. For individuals under 18 years old, consent was obtained from a parent or guardian. Participants included both patients and healthy volunteers.

Among the 191 participants, 70 visceral leishmaniasis (VL) cases were initially screened using the rK39 immunochromatographic test (ICT, InBios Int. Inc., USA) and were further confirmed parasitologically by microscopic examination of Leishman-Donovan bodies in splenic or bone marrow aspirates. Additionally, 22 post-kala-azar leishmaniasis (PKDL) cases were confirmed via PCR analysis of skin specimens. The study also included 14 cases each from endemic healthy controls (EHC) and individuals with other diseases (OD) presenting symptoms similar to VL, such as malaria (*n* = 5), viral fever (*n* = 5), typhoid (*n* = 2), and tuberculosis (*n* = 2), all obtained from hospitals. Furthermore, 47 archived non-endemic healthy controls (NEHC) samples were collected from volunteers at IICB. Follow-up (FU) samples (*n* = 24) were collected approximately six months post-treatment from VL patients. The EHC, NEHC, and OD samples were used as negative controls, while samples from VL and PKDL patients served as positive controls. FU samples were specifically analyzed to assess reactivity with antigens over time. In parallel, 40 serum samples from VL patients caused by *L. infantum* and 19 control samples were obtained from the Federal University of Piauí (UFPI) in Teresina, Brazil. These samples, collected between 2008 and 2009, were transported to India frozen and stored at − 80 °C until analysis.

The study was divided into three panels:

- Panel 1 included 184 serum samples from India: 70 from VL, 15 from PKDL, 47 from NEHC, 14 from EHC, 14 from OD, and 24 from FU.
- Panel 2 comprised 59 serum samples from Brazil: 40 from VL, 19 from HC.
- Panel 3 involved 132 urine samples from India: 57 from VL, 22 from PKDL, 23 from NEHC, 12 from EHC, 13 from OD, and 5 from FU.

Additionally, a longitudinal study was conducted with five serum and five urine samples collected from a subset of patients at three time points: before treatment (day 0), upon treatment completion (day 30), and approximately six months after treatment with AmBisome initiation (day > 180). All serum samples from India were stored at − 20 °C, while urine samples were preserved with 0.1% sodium azide and stored at 4 °C until further analysis. Graphical Abstract illustrates the antigenicity of LACK, showing its interaction with serum and urine samples from VL patients and healthy controls from endemic regions in India and Brazil.

### Ethics statements

The study received approval from the Ethical Committee on Human Subjects at CSIR-Indian Institute of Chemical Biology (Approval No. IICB/IRB/2021/I), the Institutional Ethics Committee of the School of Tropical Medicine in Kolkata (IEC Ref. No. CREC-STM/2020-AG-11), and the Rajendra Memorial Research Institute of Medical Sciences (RMRIMS) in Patna for the use of human samples. Additionally, ethically approved sera from Brazil were obtained with approval from the Ethics Committee of the Federal University of Piauí (UFPI) in Teresina, Brazil (Approval No. #116/2005).

### Cloning, overexpression and purification of LACK

The LACK gene from *L. donovani* strain AG83 (ATCCR PRA413™) was cloned into the pET28a vector and expressed in *Escherichia coli* Rosetta (DE3) cells, producing the N-terminal His-tagged LACK protein. Genomic DNA from *L. donovani* promastigotes was isolated, and the LACK gene was amplified by PCR and inserted into the pET28a (Novagen, Madison, WI, USA) vector using *Nco*I and *Hind*III restriction sites (Promega. Madison, USA). For protein overexpression, cultures were grown at 37 °C in 1 L of Luria–Bertani (HiMedia Laboratories Pvt. Ltd., Mumbai, India) medium, induced with 0.3 mmol/L isopropyl β-D-1-thiogalactopyranoside (IPTG) (Sigma-Aldrich, St. Louis, USA) when the optical density (OD) reached 0.4 to 0.6. After a 4-h induction, cells were harvested and resuspended in bacterial lysis buffer [25 mmol/L Tris-HCl, 300 mmol/L NaCl, 1 mg/ml lysozyme, and 1 mmol/L phenylmethylsulfonyl fluoride (PMSF) (Sigma-Aldrich, St. Louis, USA), pH 8.0]. The suspension was then sonicated using an ultrasonicator (Misonix, Farmingdale, NY, USA). The lysates were centrifuged at 20,130 × *g* for 30 min to separate the soluble and insoluble fractions. Inclusion bodies were solubilized in a binding buffer containing 6 mol/L urea, 10 mmol/L imidazole, and 25 mmol/L Tris-buffered saline (TBS) (Sisco Research Laboratories Pvt. Ltd., Mumbai, India) and then centrifuged at 16,639 × *g* for 30 min. The supernatants were incubated with pre-equilibrated Ni-NTA agarose resin (Qiagen, Germany) for binding of the His-tagged LACK protein. The bound protein was washed twice with urea-containing buffer [50 mmol/L imidazole, 0.1% Triton X-100], followed by two washes without Triton X-100. The recombinant LACK protein was eluted with a buffer containing 500 mmol/L imidazole (Sisco Research Laboratories Pvt. Ltd., Mumbai, India). The eluted fractions, which were denatured with urea, were dialyzed gradually to remove urea and allow proper protein folding [10]. The protein concentration was determined using the Lowry method, and the purity was confirmed by 10% SDS-PAGE followed by Coomassie Brilliant Blue staining [26]. Protein fractions were concentrated using Amicon Ultra centrifugal filter devices (Millipore Corporation, USA).

### Homology modeling

The templates for homology modeling were identified by searching for structures with maximum sequence identity using NCBI BLASTp. This tool utilizes the amino acid sequence as input to generate an alignment profile. The homology and inter-species conservation of the LACK protein were evaluated by comparing amino acid sequences from multiple *Leishmania* species, including *L. donovani, L. infantum, L. chagasi* (syn. *L. infantum*), *L. major, and L. mexicana,* as well as *Trypanosoma* species (*T. brucei* and *T. cruzi*). Multiple sequence alignments (MSA) were performed to assess conservation levels, and the results were visualized using Jalview 2.8 (University of Dundee, UK) [27].

### Immunoblot assay

Immunoblotting (Western blot) analysis for recombinant LACK antigen was performed on 10% SDS-PAGE gels, following Laemmli's method [28]. The resolved proteins were electroblotted onto a 0.45 µm nitrocellulose membrane (Bio-Rad, Germany) using a Trans-Blot apparatus (Bio-Rad, California, USA) at a constant current of 1.5 A for 15 min. Protein transfer was confirmed by Ponceau S (Sigma-Aldrich, St. Louis, USA) staining. The membrane was then cut into strips and blocked with 5% bovine serum albumin (BSA) (GOLDBIO, St. Louis, USA) in Tris-buffered saline (TBS) for 1 h. Strips were incubated overnight at 4 °C with constant shaking, using primary antibodies diluted 1:2000 from serum or 1:10 from urine samples [29]. On the following day, HRP-conjugated goat anti-human IgG secondary antibody (SouthernBiotech, Birmingham, USA) (diluted 1:3000) was added and incubated at room temperature for 1 h with shaking. The strips were washed five times with wash buffer, each wash lasting 5 min. Chemiluminescent substrate (HRP substrate, Sigma-Aldrich, USA) was then applied to the strips. Images were captured using the Bio-Rad Gel Doc system and analyzed with Image Lab software (version 5.4.2, Bio-Rad Laboratories, Inc., USA) for quantification and visualization.

### Enzyme-linked immunosorbent assay (ELISA)

To assess the performance of recombinant LACK antigen, an enzyme-linked immunosorbent assay (ELISA) was performed to measure specific IgG antibodies against the LACK antigen. Recombinant LACK (1 mg/ml) was coated onto 96-well ELISA plates (Nunc) at a volume of 100 µl per well and incubated overnight at 4 °C in 10 mmol/L phosphate buffer (PB). The following day, the plates were washed twice with phosphate-buffered saline containing 0.05% Tween 20 (PBST), and the wells were blocked with 1% bovine serum albumin (BSA) in PBS (200 µl/well) at 37 °C for 1 h. After three additional washes with PBST, serum samples (diluted 1:2000) or urine samples (diluted 1:10) containing primary antibodies were added (100 µl/well) and incubated at 37 °C for 2 h. After incubation, HRP-conjugated anti-human IgG secondary antibody (SouthernBiotech, USA) (diluted 1:3000 in PBS) was added, and the plates were incubated at 37 °C for another 2 h. The wells were then washed five times with PBST, and bound IgG was detected using 3,3',5,5'-tetramethylbenzidine (TMB) substrate (Sigma-Aldrich) [15, 16]. The reaction was terminated with 2 normal H_2_SO_2_, and optical density was measured at 450 nm using a microplate spectrophotometer (Thermo Fisher Scientific, USA).

### Dipstick preparation and assay

To develop nitrocellulose membrane-based dipsticks for field use, a membrane (2.4 × 8 cm, 0.45 µm pore size, GE Healthcare Life Sciences) was pre-soaked in 25 mmol/L Tris–HCl buffer (pH 7.6). After semi-drying, 1 µg of recombinant LACK antigen was dispensed onto the membrane at the test line, and a 1:10 dilution of rabbit anti-human IgG (unlabeled, Southern Biotech, USA) was dispensed at the control line using a Flowline F100 dispenser (Precore Solutions, Kochi, India) at a rate of 5 µl/cm. The membrane was incubated at room temperature for 30 min, followed by overnight blocking at 4 °C with a solution containing 2% bovine serum albumin (BSA), 0.1% Tween-20, and 0.01% sodium azide (NaN₃) in 100 mmol/L TBS. The membrane was then washed with TBS containing 0.05% Tween-20 (TBST). After drying, the membrane was affixed to a plastic sheet and cut into 0.4 mm diameter strips, which were stored in a desiccator for future use. For testing, the dipsticks were incubated in serum (diluted 1:2000 in TBS) or urine (diluted 1:10 in TBS) samples. After incubation, the dipsticks were treated with enzyme-conjugated anti-human HRP-IgG (Southern Biotech, diluted 1:2000). The strips were washed twice with TBST and once with TBS. The colorimetric reaction was developed by immersing the strips in a substrate solution containing 0.05% 3,3'-diaminobenzidine tetrahydrochloride (DAB, Sigma-Aldrich, USA) and 0.05% hydrogen peroxide (H_2_O_2_, MP Biomedicals, France) in 100 mmol/L TBS. The reaction was stopped by dipping the strips in distilled water. The appearance of dark brown bands at both the test and control lines indicated a positive result for VL, while a single band at the control line indicated a negative result [30].

### Data analysis

Receiver operating characteristic (ROC) curves and statistical analyses were performed using GraphPad Prism 8.0 software (Boston, USA). ROC curves were constructed with 95% confidence intervals, comparing VL and HC samples to identify thresholds that maximized sensitivity and specificity. Sensitivity was defined as the percentage of VL samples correctly identified as positive, while specificity was calculated as the percentage of non-VL samples (including NEHC, EHC, and OD) correctly identified as negative. The area under the curve (AUC) was used to evaluate the diagnostic accuracy of each test, with a higher AUC indicating better performance, and an AUC of 1 representing perfect accuracy. Statistical significance was determined using the Mann–Whitney U test, with a *P* value of < 0.05 considered significant [31].

## Results

### Identification, sequence analysis, cloning and purification of *L. donovani* LACK

**Fig. 1:**
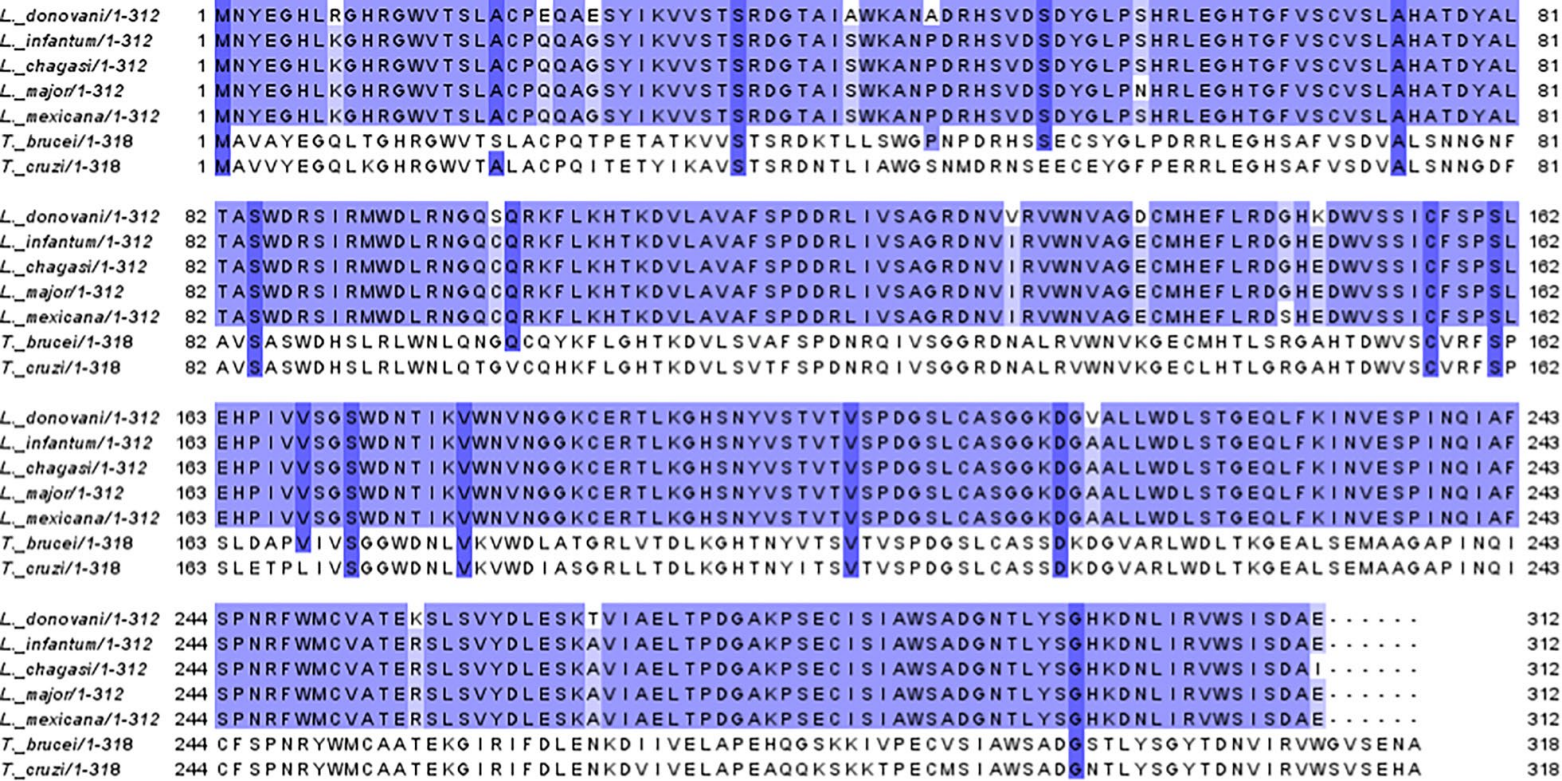
Sequence Comparison of LACK (LdBPK_282970.1). Multiple sequence alignment of amino acid sequences was performed using Jalview 2.8 to compare LACK homologs from *L. donovani* (BAB91559.1), *L. infantum* (XP_001470319.2), *L. chagasi (*syn. *L. infantum)* (ABS82039.1), *L. major* (XP_001684560.1), *L. mexicana* (XP_003877119.1), *T. brucei* (XP_011780414.1), and *T. cruzi* (XP_817733.1) obtained from NCBI database. Conserved residues are highlighted with a purple background, with the intensity of the color reflecting the degree of conservation. The numbers on the left and right indicate the positions of the amino acids

**Fig. 2:**
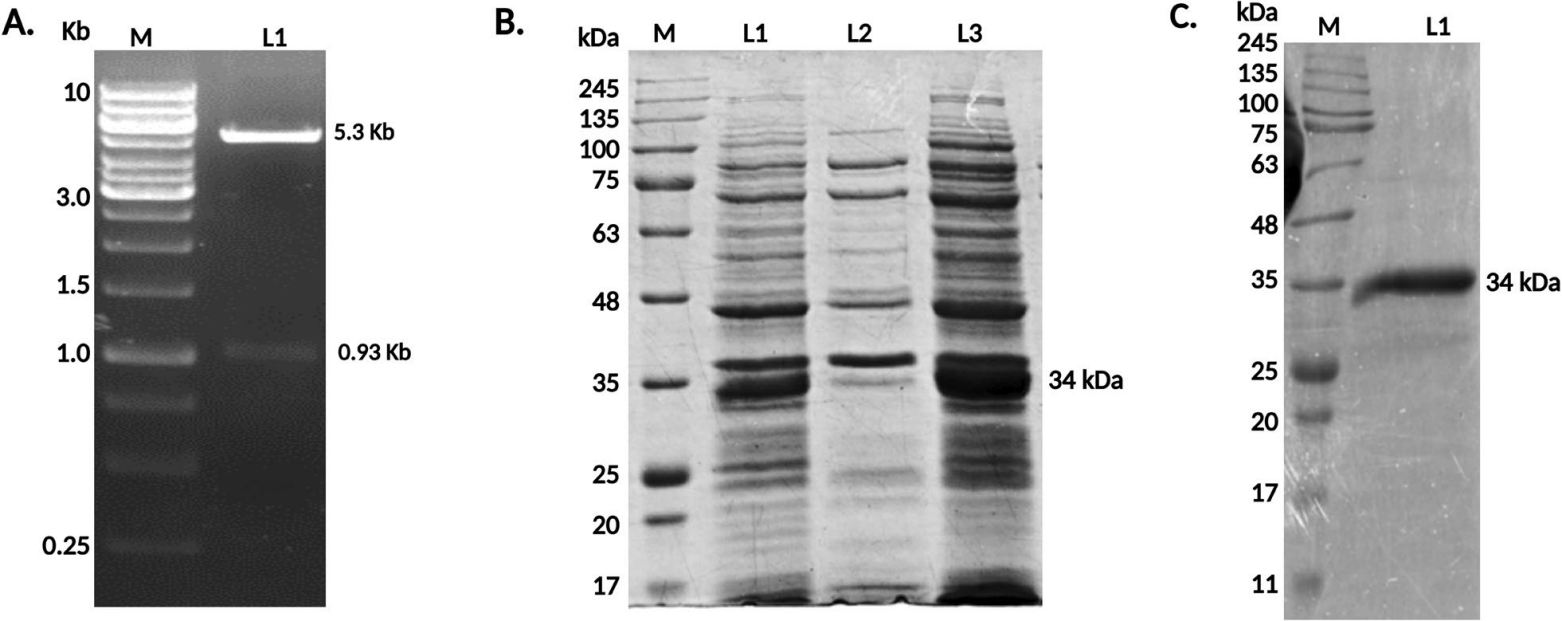
Cloning and expression of purified recombinant *L. donovani* LACK. (**A**) LACK was PCR amplified and cloned into pET28a. The marker (M) shows base pair sizes, while lanes (L1) display PCR products of 5.3 kb (pET28a) and 0.93 kb (LACK), respectively. (**B**) Following IPTG induction, LACK expression resulted in a 34 kDa protein, indicated by lanes 1 and 3 (lysates from cultures induced with 0.3 mmol/L IPTG), lane M (molecular weight markers), lane 2 (uninduced lysate), and (**C**) The purification of LACK is shown, in lane 1 presenting the recombinant protein from the eluted fraction

A complete open reading frame (ORF) of the *L. donovani* LACK (LdBPK_282970.1) gene, with a length of 939 bp, was retrieved from the triTrypDB database. This gene encodes a protein of 312 amino acids, with an approximate molecular weight of 34 kDa. In the MSA, the homology and inter-species conservation of the LACK protein among various *Leishmania* and *Trypanosoma* species were highlighted in purple, with color intensity reflecting the degree of conservation (Fig. 1). The analysis revealed that LACK is highly conserved among *Leishmania* species but shows lower conservation in *Trypanosoma* and no detectable conservation in humans. Amino acid sequences for the proteins were retrieved from the NCBI database for the BPK strain of *L. donovani*, followed by a BLAST search of the genomic databases for visceralizing *Leishmania* species. This yielded five strains as top scorers based on sequence similarity (Table 1). LACK demonstrated a high degree of sequence identity (98.72–100%) with strains of *L. infantum* from Iran and Spain, as well as *L. donovani* strains from Sudan, and *L. chagasi (*syn. *L. infantum)* from Brazil. During cloning, PCR amplification was performed using LACK gene-specific primers on the genomic DNA of *L. donovani* strain AG83. The resulting product, which corresponded to a gene size of LACK, was successfully cloned. A 0.93 kb band was observed in the fall-out experiment (Fig. 2A), confirming the successful cloning of the gene. The recombinant LACK protein was then expressed in *E. coli* Rosetta (DE3) strain by induction with IPTG (Fig. 2B). Expression of LACK protein was maximal in lanes 1 and 3, relative to the uninduced sample in lane 2. Finally, SDS-PAGE analysis of the purified recombinant protein revealed a band corresponding to the expected molecular weight of 34 kDa in the elution fractions (Fig. 2C).

**Table 1:** GenBank sequence comparison of LACK within *Leishmania* strains causing visceral leishmaniasis

Gene and accession number	Strain	Scientific name	Origin	Base pair (kb)	Protein length (aa)	Accession	Percent identity (%)
*Leishmania donovani* activated protein kinase c receptor (LACK) (LdBPK_282970.1)	MCAN/IR/10/Resist1	*L. infantum*	Iran	939	312	JX305921.1	98.72
	MCAN/ES/98/LLM-724 (JPCM5)	*L. infantum*	Spain	939	312	XM_001470282.2	100
	MHOM/SD/001S-2D p36	*L. donovani*	Sudan	939	312	AF363974.1	99.79
	MHOM/IN/80/Dd8	*L. donovani*	India	939	312	XM_003862346.1	100
	MHOM/BR/73/M2269	*L. infantum*	Brazil	936	312	EU016105.1	99

### Immunoblot assay based LACK validation with serum and urine samples from India

**Fig. 3:**
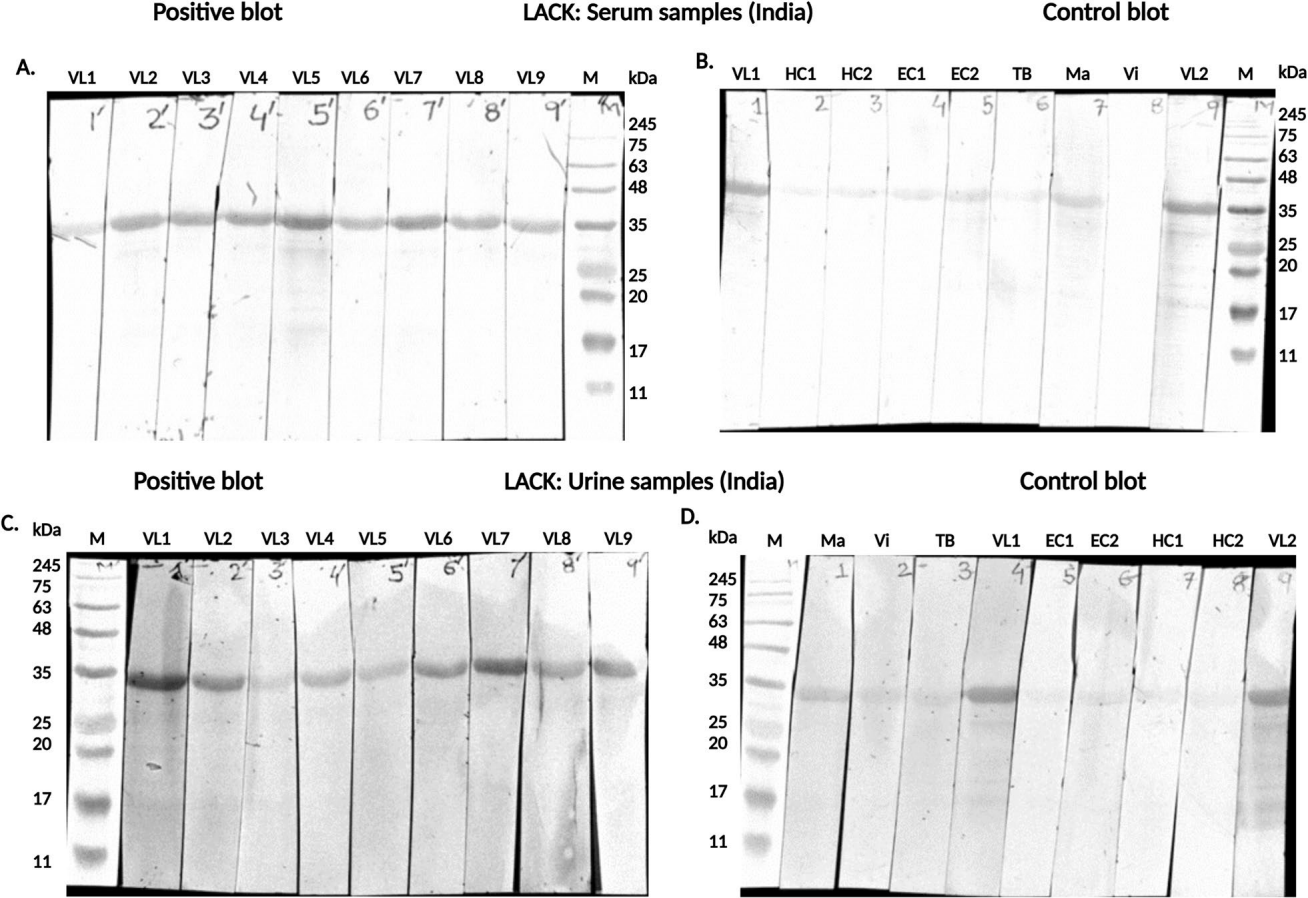
Immunoblot assay of recombinant *Leishmania* protein LACK. **A** In positive immunoblot LACK was tested with 9 VL positive serum samples (VL1 to 9), with protein markers in lane M. **B** In control immunoblot LACK carried out with non-endemic healthy controls in lanes 2 to 3 (HC1 and HC2), endemic healthy controls in lanes 4 to 5 (EC1 and EC2), other diseases in lanes 6, 7 and 8, including tuberculosis (TB), malaria (Ma) and viral fever (Vi), respectively and VL-positive sample in lanes 1 and 9, protein marker in lane M. **C** In another set of experiment, positive immunoblot assay of recombinant LACK with 9 VL urine samples (VL1 to 9), with protein marker in lane M. **D** Control immunoblot assays of LACK was done with non-endemic healthy controls in lanes 7 and 8 (HC1 and HC2), endemic healthy controls in lanes 5 and 6 (EC1, and EC2), and other diseases in lanes 1 to 3, including malaria (Ma), viral fever (Vi) and tuberculosis (TB), respectively, VL-positive samples in lane 4 and lane 9, with protein markers in lane (M)

The reactivity of recombinant LACK antigen was evaluated by four immunoblots using serum and urine from VL-positive patients and healthy controls. LACK showed strong reactivity with VL patient samples, consistently displaying a 34 kDa band. (Fig. 3A and C). In the control serum assay (Fig. 3B), the LACK antigen did not bind to serum from endemic or non-endemic healthy individuals, nor to samples from diseases similar to VL, except for a faint cross-reaction with the malaria sample (lane 1) and positive binding with the VL patient samples (lanes 1 and 9). In the urine control assay (Fig. 3D), the antigen showed no reactivity with serum from non-endemic or endemic healthy controls (lanes 5 to 8) and no binding with urine from other diseases (TB, viral fever, malaria), except for weak reactivity with the VL samples (lanes 4 and 9) and the malaria sample (lane 1).

### Performance of LACK based ELISA using serum samples from India and Brazil

**Fig. 4:**
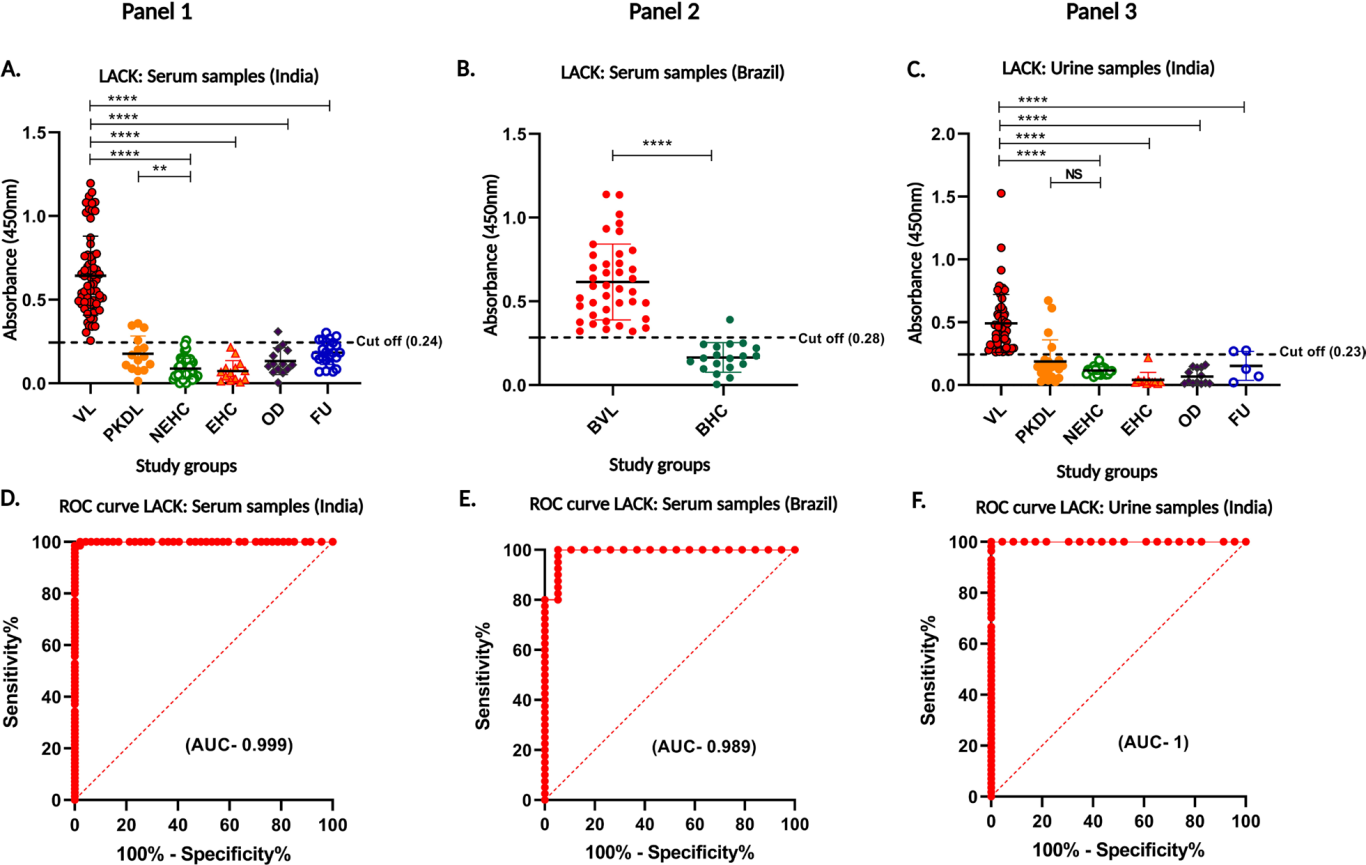
ELISA with recombinant kinase antigen LACK. Panel 1 (**A**) The sera for this study were collected from Indian VL patients (VL, *n* = 70), post kala-azar dermal leishmaniasis patients (PKDL, *n* = 16), non-endemic healthy controls (NEHC, *n* = 47), endemic healthy controls (EHC, *n* = 14), other diseases (OD, *n* = 14), and follow-up patients (FU, *n* = 24). Panel 2 (**B**) The sera were collected from Brazilian VL patients (BVL, *n* = 40), and healthy controls (BHC, *n* = 19). Panel 3 (**C**) The urine samples for this study were collected from Indian VL patients (VL, *n* = 57), post kala-azar dermal leishmaniasis (PKDL, *n* = 22), non-endemic healthy controls (NEHC, *n* = 23), endemic healthy controls (EHC, *n* = 12), other diseases (OD, *n* = 13), and follow-up patients (FU, *n* = 5). The dotted horizontal lines represent the cutoff values (0.24, 0.28 and 0.23) for each ELISA from Figure A, B and C respectively, calculated using ROC curves where the highest sensitivity and specificity were obtained. (****P < 0.0001). **D**, **E** and **F** ROC curves obtained from ELISA using LACK antigen for detection of antigen-specific antibodies in serum samples

After confirming the immunogenicity of the LACK antigen in the immunoblot assay, we expanded the study to include a larger, more diverse sample set for the full ELISA-based assay. The study was divided into two panels, with a comprehensive collection of serum samples from VL-positive patients in India (Panel 1) and Brazil (Panel 2). The cutoff values for the ELISA assay were set at 0.24 for the Indian samples and 0.28 for the Brazilian samples. The area under the curve (AUC) values were 0.99 for Indian VL sera and 0.98 for Brazilian VL sera. The sensitivity was 100% for both the Indian and Brazilian VL cases, with 95% confidence intervals (*CI*s) of 94.80–100% for India and 91.24–100% for Brazil, confirming that all VL cases were correctly identified. Specificity was 97.33% (95% *CI*: 90.79–99.53%) for the Indian control group and 94.74% (95% *CI*: 75.36–99.73%) for the Brazilian control group (Fig. 4A and B, Table 2). However, the LACK-ELISA assay showed a lower sensitivity for detecting PKDL cases. Only 4 PKDL samples were above the cutoff, resulting in a sensitivity of 26.67%. Statistical analysis using the Mann–Whitney U test and ROC curve calculations for Indian and Brazilian VL sera compared to healthy controls are shown in Fig. 4D and E, respectively, further supporting the diagnostic utility of the LACK antigen for VL detection.

**Table 2:** Performance of *Leishmania donovani*–LACK in the diagnosis of Indian and Brazilian visceral leishmaniasis cases

		Samples, No./Total, 95% confidence interval								
Country and test	Sample type	VL	PKDL	NEHC	EHC	OD	Total control	VL follow-up	AUC value	Cut off value
INDIA		(Sensitivity, %)		(Specificity, %)				(Reactivity, %)		
ELISA	Serum	100	26.67	97.87	100	92.86	97.33	20.83	0.999	0.24
		(70/70)	(4/15)	(46/47)	(14/14)	(13/14)	(73/75)	(5/24)		
		94.80–100	10.90**–**51.95	88.89**–**99.89	78.47**–**100	68.53**–**99.63	90.79–99.53	…		
	Urine	100	22.73	100	100	100	100	40	1	0.23
		(57/57)	(5/22)	(23/23)	(12/12)	(13/13)	(48/48)	(2/5)		
		94.80–100	10.12**–**43.44	85.69**–**100	74.12**–**100	77.19**–**100	…	…		
DIPSTICK	Serum	100	26.67	100	100	100	100	20	…	…
		(20/20)	(4/15)	(20/20)	(10/10)	(10/10)	(40/40)	(2/10)		
	Urine	100	20	100	100	100	100	20	…	…
		(20/20)	(3/15)	(20/20)	(10/10)	(10/10)	(40/40)	(1/5)		
BRAZIL										
ELISA	Serum	100	…	94.74	…	…	94.74	…	0.989	0.28
		(40/40)		(18/19)			(18/19)			
		91.24–100		75.36**–**99.73			75.36–99.73			
DIPSTICK	Serum	100	…	100	…	…	100	…	…	…
		(20/20)		(15/15)			(15/15)			

… means not applicable

*EHC* endemic healthy controls, *NEHC* non-endemic healthy controls, *OD* other diseases, *FU* follow up patients, *AUC* area under the curve

### Performance of LACK based ELISA for detection of urine IgG antibodies in Indian VL patients

In our investigation, we extended the assessment of antigen-specific antibodies against LACK to urine samples (Panel 3). ROC curves were employed to establish cutoff values (0.23) and AUC value of 1 indicating the antigen's performance in detecting VL. ELISA analysis revealed excellent reactivity of the antigen with VL antibodies in urine, exhibiting a sensitivity of 100% (95% *CI*: 94.80–100%), and a specificity of 100% (95% *CI*: 92.44–100%) including combined non-endemic, endemic healthy control and other disease urine samples (Fig. 4C). Moreover, in the case of PKDL, 5 positive samples out of 22 were above the cutoff line, resulting in a sensitivity of 22.73%. Using Mann–Whitney U test, the ROC curves obtained for LACK are shown in Fig. 4F, comparing VL sera against with healthy control sera.

### Utility of LACK antigen detection ELISA test in monitoring VL treatment

**Fig. 5:**
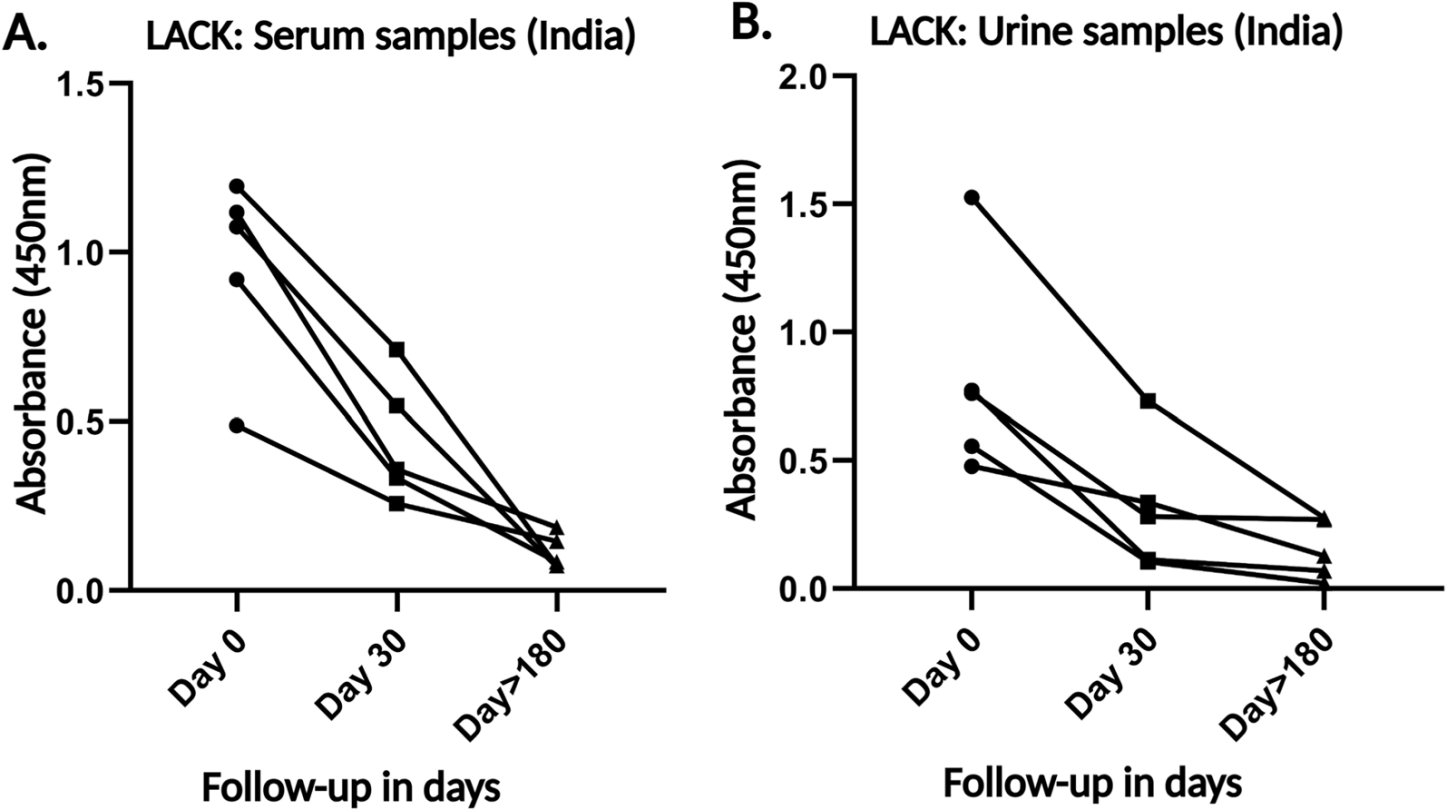
Comparison of levels of antibodies in (**A**) serum (1∶2000 dilution) and (**B**) urine (1∶10 dilution) of VL patients before treatment started and post-treatment. Absorbance values of antibodies specific to before the treatment of VL started at day 0, 1 month after treatment at day 30, and at least 6 months after treatment at day 180. Paired t tests were done to compare the antibodies at different time points

To evaluate the treatment response, we analyzed the presence of antigen-specific antibodies against LACK in serum and urine samples from Indian follow-up (FU) cases. ELISA results, analyzed using the Mann–Whitney U test, showed a significant decrease in antigen positivity in both serum and urine samples at the follow-up stage, with reactivity observed in only 20.83% of the samples (Fig. 4A and C). This is in contrast to the higher levels of VL-specific antibodies observed before treatment. A small number of FU samples did exceed the cutoff values—0.24 for serum and 0.23 for urine—but overall showed minimal reactivity, indicating a reduction in antigen-specific antibody levels (Table 2). For a more in-depth analysis of treatment response, paired serum (Fig. 5A) and urine (Fig. 5B) samples from five *Leishmania*-infected patients were tested at three time points: before treatment, 1 month after treatment, and at least 6 months post-treatment. Antibody levels against the LACK antigen showed a sharp decline after 30 days of treatment, continuing to decrease to very low levels by 180 days post-treatment.

### Performance of LACK based dipstick assay with serum and urine samples from India and serum samples from Brazil

**Fig. 6:**
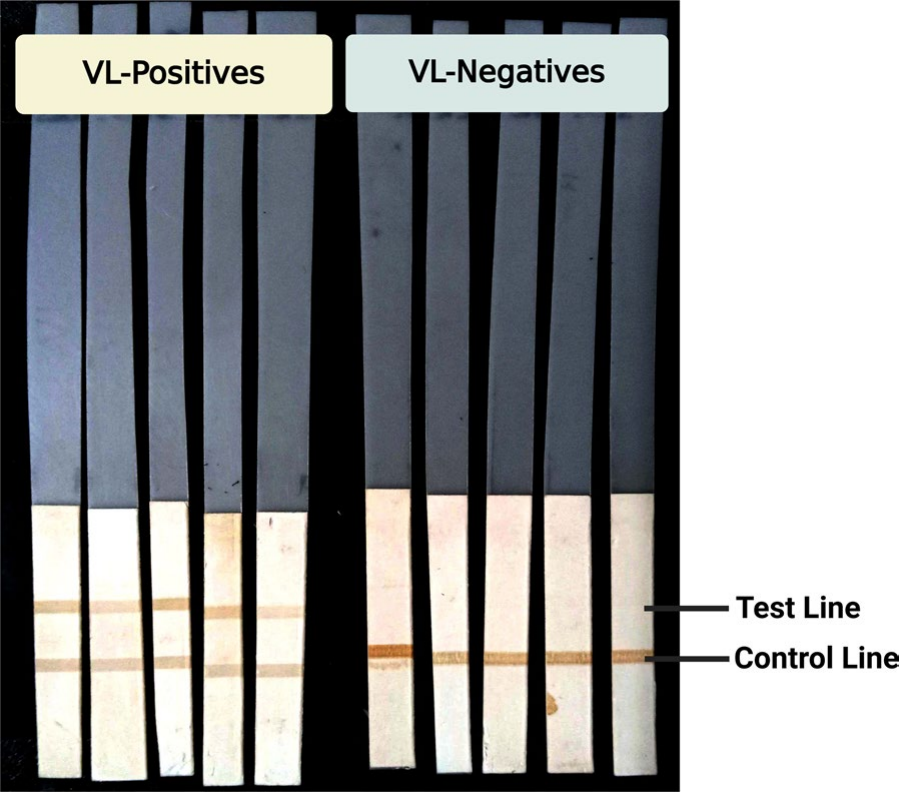
Representative image of dipstick immunochromatographic test shows a positive result with two bands (test and control) and a negative result with a single band at the control line

After confirming the immunogenicity of the LACK antigen through immunoblot and ELISA assays, we evaluated its diagnostic potential in field-adaptable settings using a dipstick assay. VL serum samples from both India and Brazil produced strong, dark bands appeared in both the test and control lines on the nitrocellulose membrane, resulting in a sensitivity of 100%. For the negative controls from India (NEHC, EHC, and OD), all bands appeared only in the control region, with no cross-reactivity in the test line. This resulted in a specificity of 100% for the Indian control samples. Similarly, the Brazilian samples also showed 100% specificity against healthy controls. In addition, when tested with Indian VL urine samples, the dipstick assay achieved 100% sensitivity and specificity. FU samples showed reduced reactivity with the LACK antigen, with only 20% of the treated samples showing positive results (Fig. 6). Table 2 compares the analytical performance of the LACK dipstick test with that of the ELISA.

## Discussion

This study addresses the challenge of developing a reliable diagnostic tool by analyzing samples from India and Brazil. While rK39-based rapid tests show promising results in India, they exhibit variable sensitivity and specificity in Africa and Brazil and are unsuitable for post-treatment monitoring [32, 33]. We identified, cloned, and purified a highly conserved kinase protein, LACK, and, for the first time, assessed its serological performance using VL serum samples from India and Brazil, as well as Indian VL urine samples. Immunological assays, including western blot, ELISA, and dipstick tests, were employed to evaluate its potential. Our lab had previously reported a range of electroeluted proteins from *L. donovani* membrane antigens (LAg) via MALDI-TOF mass spectrometry, identifying the 34 kDa LACK protein by matching it with the *Leishmania* sequence and further assessed its protective efficacy against VL [34]. LACK, a highly conserved kinase antigen among *Leishmania* species with no sequence homology to humans, has been proposed as a promising vaccine candidate for both cutaneous and visceral leishmaniasis [35]. Fernandez et al. reported that prime-boost vaccination with pCI-neo-LACK/MVA-LACK protects against *L. infantum*, highlighting LACK's potential for VL vaccine development [36]. Our results demonstrated strong and specific recognition of human anti-*L. donovani* IgG antibodies in Indian samples and anti-*L. infantum* IgG antibodies in Brazilian samples. Given its role in virulence across different *Leishmania* species and parasite stages, we were particularly interested in examining the sequence similarity between LACK from various visceralizing *Leishmania* species in endemic regions worldwide. Using NCBI BLAST, we found that LACK shares 98.72% to 100% sequence identity with strains of *L. infantum* from Iran and Spain, as well as *L. donovani* strains from Sudan, India, and *L. chagasi* from Brazil. This bioinformatic analysis supports the potential of LACK as a cross-regional diagnostic antigen for both *L. donovani* and *L. infantum*, which are responsible for visceral leishmaniasis in different endemic areas around the world. The significance of kinases in VL diagnosis was further emphasized by Marlais et al. (2020), who identified kinase proteins (LdBPK_351070) from Indian and Sudanese urine samples via mass spectrometry and analyzed epitope regions contributing to the development of non-invasive immunoassays for this deadly disease [37].

Currently, several kinesin-based antigens, including rK28, rK26, rKRP42, rKE16, rKLO8, and rKLi8.3, have been developed for use in immunological assays such as ELISA, dipstick tests, and rapid diagnostic tests (RDTs) to detect antibodies in patients with VL in endemic regions [33, 38–40]. Notably rK39 and rK28, have been validated using VL samples from endemic areas across the Americas, East Africa, and the Indian subcontinent, with varying performance outcomes [41, 42]. rK39-based serum RDTs show 97% sensitivity in the Indian subcontinent. However, sensitivity drops to 85% in East Africa and ranges from 88 to 94% in Brazil [43, 44]. Furthermore, the rK39 urine-based variant shows 96% sensitivity but only 68% specificity, which poses challenges in regions like India, where its low specificity may lead to cross-reactivity with other diseases such as malaria and enteric fever [38, 45]. This cross-reactivity can result in misdiagnosis and inappropriate treatment. Our study investigating the kinase-based recombinant antigen, LACK, commenced with pilot experiments using membrane-based Western blot assays to assess the antigen's reactivity with antibodies in VL samples. This initial validation involved a small sample size, consisting of 18 Indian patient samples from both serum and urine, to confirm the feasibility and specificity of the antigen. Testing LACK against these serum and urine samples demonstrated strong reactivity with VL samples, while showing only mild reactivity with control samples. Additionally, the LACK ELISA demonstrated excellent performance, achieving 100% sensitivity in detecting antibodies from *Leishmania*-infected serum samples not only from India but also from Brazil. However, the specificity was lower in Brazil due to some reactivity with antigens, particularly with serum negative controls. Notably, compared to 2023 data by Fujimori et al., LACK-ELISA (100%) showed significantly higher sensitivity in VL samples from Brazil than rK18-ELISA (83.3%), rKR95-ELISA (95.6%), rK39-ELISA (94.3%), and rK28-ELISA (95.9%). These findings suggest that LACK antigen is a promising alternative for diagnosing human VL in Brazil [44].

In clinical settings, blood sampling can be challenging, especially for malnutrition young children who may be reluctant to provide blood samples [46]. Non-invasive specimens, such as urine, offer an attractive alternative for diagnosis due to their easier collection and storage compared to serum. KAtex, a well-established urine antigen capture assay, detects a carbohydrate antigen with 84–100% specificity but lower sensitivity (47–87%) and requires boiling urine samples before testing, limiting its practicality [47]. We first time optimized the LACK ELISA for use with VL urine samples from India and found it demonstrated 100% sensitivity for VL and 100% specificity with negative control samples. Importantly, LACK did not detect antibodies in PKDL patients from India. Serum- and urine-based ELISA results for PKDL patients were not significantly different from those of non-endemic healthy controls, suggesting lower sensitivity for PKDL while highlighting LACK's ability to robustly distinguish between VL and PKDL infections.

We further explored the transition from ELISA to a dipstick assay, which holds promise for field-based diagnostics. This assay proved to be user-friendly and significantly reduced the diagnostic time from 8 h ELISA to just 2 h. When evaluated with both serum and urine samples from VL patients and healthy controls, the LACK dipstick showed clear positivity in VL samples with 100% sensitivity and no reactivity with negative controls, including samples from healthy individuals in endemic and non-endemic regions, as well as samples from other diseases. The dipstick assay also exhibited 100% specificity, outperforming the ELISA in terms of affinity to the samples. When we tested the LACK-based dipstick using serum samples from Brazil, it showed clear positivity in VL cases, achieving 100% sensitivity and specificity. This suggests that the LACK-based ELISA and dipstick could serve as valuable diagnostic tools for VL caused by *L. infantum*.

In areas where VL is endemic, antibodies against the parasite can persist long after an infection, either due to repeated exposure to *L. donovani* or incomplete eradication of the parasite [48]. Our longitudinal study revealed a significant decrease in LACK-specific antibody levels in both serum and urine samples 180 days post-treatment. This decline was consistent with findings from the dipstick assay, which showed a 20% antibody reactivity to LACK six months post-treatment. In contrast, the rK39 test retained 86.66% reactivity even after six months [17], highlighting the advantage of LACK in monitoring treatment progress. To further evaluate antibody dynamics over time, measuring immunoglobulin G1 (IgG1) responses to LACK could provide deeper insights. A recent study demonstrated that IgG1 detection against rK39 is useful for monitoring VL treatment outcomes, as IgG1 levels tend to decline rapidly in the absence of a continuous antigenic stimulus [49]. Overall, our results underscore the potential of LACK-based ELISA and dipstick assays for both VL diagnosis and treatment monitoring. These assays offer significant advantages over existing diagnostics in terms of specificity and the ability to distinguish between different disease stages. Additionally, the dipstick proves to be a promising tool for affordable and rapid VL diagnosis in field settings. However, further longitudinal studies are needed to fully understand its prognostic potential.

While our findings are promising, there are several limitations to this study. We focused solely on clinically confirmed VL cases, limiting our ability to evaluate the antigen’s performance in suspected VL cases. Additionally, although we assessed clinically confirmed VL cases across different age groups using ELISA and dipstick assays, we did not analyze age-wise reactivity of IgG antibodies with the LACK antigen. Samples from patients with other VL-related conditions or those co-infected with HIV were also excluded. Further studies with larger Brazilian sample sizes, including VL-negative samples from individuals with varying immunological backgrounds (e.g., Chagas disease), are necessary to confirm these results. Furthermore, establishing an optimal cut-off value for ELISA-based assays remains challenging, as it requires balancing sensitivity and specificity, particularly when absorbance values are near the cut-off. Continued research is needed to refine the LACK-based rapid diagnostic test for faster, field-adaptable diagnostics.

Despite these limitations, the LACK antigen demonstrated encouraging results in this preliminary study, showing potential for rapid VL diagnosis and monitoring of treatment outcomes. These findings support the need for further investigation in the next phase of research, which should include LACK-based assays with larger and more diverse set of samples from defined VL cases and controls, particularly from regions in Africa where the disease burden is high. Additionally, further research should focus on geographically diverse cohorts, optimization for field adaptability, and the establishment of standardized cut-off values to enhance diagnostic accuracy. These efforts will be essential in transitioning the LACK-based diagnostic approach from preliminary research to a widely applicable and robust tool for VL diagnosis and treatment monitoring. Developing effective diagnostic tools for VL is critical not only for controlling this neglected tropical disease but also for advancing the United Nations' Sustainable Development Goal of achieving Universal Health Coverage [50].

## Conclusions

Our study highlights the promising potential of the LACK antigen as a reliable and cross-regional diagnostic tool for VL. The antigen demonstrated high sensitivity and specificity in detecting anti-*Leishmania* antibodies in both serum and urine samples from VL patients in India and serum samples from Brazil. The optimized LACK ELISA and dipstick assays showed robust performance, outperforming existing diagnostic methods, particularly in distinguishing between VL and PKDL infections. Additionally, with a limited number of follow-up samples, LACK exhibited the potential to monitor treatment progress, as antibody levels decreased significantly post-treatment; however, this requires confirmation through the assessment of a larger follow-up sample set. These findings suggest that LACK-based diagnostics could provide a valuable alternative to current tools, addressing the challenges of regional variability, sensitivity, and non-invasive sample collection. Further validation in larger, more diverse patient cohorts, particularly in African endemic regions, is essential to confirm its widespread applicability and to advance VL diagnosis and management globally.

## Data Availability

All the information provided in the research is contained within the article. For additional information, please contact the corresponding author.

## References

[1] Scarpini S, Dondi A, Totaro C, Biagi C, Melchionda F, Zama D, et al. Visceral leishmaniasis: epidemiology, diagnosis, and treatment regimens in different geographical areas with a focus on pediatrics. Microorganisms. 2022;10(10):1887. 36296164 10.3390/microorganisms10101887 PMC9609364

[2] Mann S, Frasca K, Scherrer S, Henao-Martinez AF, Newman S, Ramanan P, et al. A review of leishmaniasis: current knowledge and future directions. Curr Trop Med Rep. 2021;8(2):121–32. 33747716 10.1007/s40475-021-00232-7 PMC7966913

[3] Kumar Vaitheeswaran K, Gupta BK, Krishnan GR, Soneja M, Vikram NK, Baitha U, et al. Neuro-leishmaniasis with cauda equina syndrome and cranial nerve palsy: a rare manifestation of recurrent atypical visceral leishmaniasis. BMC Infect Dis. 2024;24(1):1253. 39506665 10.1186/s12879-024-10082-z PMC11539647

[4] Hasan MM, Proma SB, Hossain MS, Arifuzzaman M, Islam N, Siddique MAB, et al. A case report on para-kala-azar dermal leishmaniasis: an unresolved mystery. BMC Infect Dis. 2023;23(1):885. 38110894 10.1186/s12879-023-08918-1 PMC10729440

[5] Reimao JQ, Coser EM, Lee MR, Coelho AC. Laboratory diagnosis of cutaneous and visceral leishmaniasis: current and future methods. Microorganisms. 2020;8(11):1632. 33105784 10.3390/microorganisms8111632 PMC7690623

[6] Tekle E, Dese K, Girma S, Adissu W, Krishnamoorthy J, Kwa T. DeepLeish: a deep learning based support system for the detection of leishmaniasis parasite from Giemsa-stained microscope images. BMC Med Imaging. 2024;24(1):152. 38890604 10.1186/s12880-024-01333-1 PMC11186139

[7] Pagliano P, Ascione T, Di Flumeri G, Boccia G, De Caro F. Visceral leishmaniasis in immunocompromised: diagnostic and therapeutic approach and evaluation of the recently released IDSA guidelines. Infez Med. 2016;24(4):265–71. 28011960

[8] Leveque MF, Battery E, Delaunay P, Lmimouni BE, Aoun K, L’Ollivier C, et al. Evaluation of six commercial kits for the serological diagnosis of Mediterranean visceral leishmaniasis. PLoS Negl Trop Dis. 2020;14(3): e0008139. 32210438 10.1371/journal.pntd.0008139 PMC7135331

[9] Ndao M. Diagnosis of parasitic diseases: old and new approaches. Interdiscip Perspect Infect Dis. 2009;2009: 278246. 20069111 10.1155/2009/278246 PMC2804041

[10] Bhattacharyya A, Kamran M, Ejazi SA, Das S, Didwania N, Bhattacharjee R, et al. Revealing a novel antigen repressor of differentiation kinase 2 for diagnosis of human visceral leishmaniasis in India. Pathogens. 2022;11(2):120. 35215064 10.3390/pathogens11020120 PMC8879085

[11] Soroka M, Wasowicz B, Rymaszewska A. Loop-mediated isothermal amplification (LAMP): the better sibling of PCR? Cells. 2021;10(8):1931. 34440699 10.3390/cells10081931 PMC8393631

[12] Ejazi SA, Choudhury ST, Bhattacharyya A, Kamran M, Pandey K, Das VNR, et al. Development and clinical evaluation of serum and urine-based lateral flow tests for diagnosis of human visceral leishmaniasis. Microorganisms. 2021;9(7):1369. 34201902 10.3390/microorganisms9071369 PMC8305891

[13] Hagos DG, Schallig H, Kiros YK, Abdulkadir M, Wolday D. Performance of rapid rk39 tests for the diagnosis of visceral leishmaniasis in Ethiopia: a systematic review and meta-analysis. BMC Infect Dis. 2021;21(1):1166. 34789175 10.1186/s12879-021-06826-w PMC8600897

[14] Abass E, Kang C, Martinkovic F, Semiao-Santos SJ, Sundar S, Walden P, et al. Heterogeneity of *Leishmania**donovani* parasites complicates diagnosis of visceral leishmaniasis: comparison of different serological tests in three endemic regions. PLoS ONE. 2015;10(3): e0116408. 25734336 10.1371/journal.pone.0116408 PMC4348478

[15] Ejazi SA, Bhattacharya P, Bakhteyar MA, Mumtaz AA, Pandey K, Das VN, et al. Noninvasive diagnosis of visceral leishmaniasis: development and evaluation of two urine-based immunoassays for detection of *Leishmania**donovani* Infection in India. PLoS Negl Trop Dis. 2016;10(10): e0005035. 27741241 10.1371/journal.pntd.0005035 PMC5065134

[16] Saha S, Goswami R, Pramanik N, Guha SK, Saha B, Rahman M, et al. Easy test for visceral leishmaniasis and post-Kala-azar dermal leishmaniasis. Emerg Infect Dis. 2011;17(7):1304–6. 21762596 10.3201/eid1707.100801 PMC3381407

[17] Kamran M, Ejazi SA, Choudhury ST, Bhattacharyya A, Tanishka K, Pandey K, et al. A novel antigen, otubain cysteine peptidase of *Leishmania**donovani*, for the serodiagnosis of visceral leishmaniasis and for monitoring treatment response. Clin Infect Dis. 2021;73(7):1281–3. 33987660 10.1093/cid/ciab435

[18] Baker N, Catta-Preta CMC, Neish R, Sadlova J, Powell B, Alves-Ferreira EVC, et al. Systematic functional analysis of *Leishmania* protein kinases identifies regulators of differentiation or survival. Nat Commun. 2021;12(1):1244. 33623024 10.1038/s41467-021-21360-8 PMC7902614

[19] Efstathiou A, Smirlis D. *Leishmania* protein kinases: important regulators of the parasite life cycle and molecular targets for treating leishmaniasis. Microorganisms. 2021;9(4):691. 33801655 10.3390/microorganisms9040691 PMC8066228

[20] Gomez-Arreaza A, Acosta H, Barros-Alvarez X, Concepcion JL, Albericio F, Avilan L. *Leishmania**mexicana*: LACK (*Leishmania* homolog of receptors for activated C-kinase) is a plasminogen binding protein. Exp Parasitol. 2011;127(4):752–61. 21272581 10.1016/j.exppara.2011.01.008

[21] Kelly BL, Stetson DB, Locksley RM. *Leishmania**major* LACK antigen is required for efficient vertebrate parasitization. J Exp Med. 2003;198(11):1689–98. 14657221 10.1084/jem.20031162 PMC2194132

[22] Julia V, Rassoulzadegan M, Glaichenhaus N. Resistance to *Leishmania**major* induced by tolerance to a single antigen. Science. 1996;274(5286):421–3. 8832890 10.1126/science.274.5286.421

[23] Perez-Jimenez E, Kochan G, Gherardi MM, Esteban M. MVA-LACK as a safe and efficient vector for vaccination against leishmaniasis. Microbes Infect. 2006;8(3):810–22. 16504562 10.1016/j.micinf.2005.10.004

[24] Costa Souza BLS, Pinto EF, Bezerra IPS, Gomes DCO, Martinez AMB, Re MI, et al. Crosslinked chitosan microparticles as a safe and efficient DNA carrier for intranasal vaccination against cutaneous leishmaniasis. Vaccine X. 2023;15: 100403. 38026045 10.1016/j.jvacx.2023.100403 PMC10665653

[25] Alonso A, Alcolea PJ, Larraga J, Peris MP, Esteban A, Cortes A, et al. A non-replicative antibiotic resistance-free DNA vaccine delivered by the intranasal route protects against canine leishmaniasis. Front Immunol. 2023;14:1213193. 37790927 10.3389/fimmu.2023.1213193 PMC10543895

[26] Lowry OH, Rosebrough NJ, Farr AL, Randall RJ. Protein measurement with the Folin phenol reagent. J Biol Chem. 1951;193(1):265–75. 14907713

[27] Waterhouse AM, Procter JB, Martin DM, Clamp M, Barton GJ. Jalview Version 2–a multiple sequence alignment editor and analysis workbench. Bioinformatics. 2009;25(9):1189–91. 19151095 10.1093/bioinformatics/btp033 PMC2672624

[28] Laemmli UK. Cleavage of structural proteins during the assembly of the head of bacteriophage T4. Nature. 1970;227(5259):680–5. 5432063 10.1038/227680a0

[29] Didwania N, Ejazi SA, Chhajer R, Sabur A, Mazumder S, Kamran M, et al. Evaluation of cysteine protease C of *Leishmania**donovani* in comparison with glycoprotein 63 and elongation factor 1alpha for diagnosis of human visceral leishmaniasis and for posttreatment follow-up response. J Clin Microbiol. 2020;58(11):e00213-e220. 32848039 10.1128/JCM.00213-20 PMC7587114

[30] Ejazi SA, Ghosh S, Saha S, Choudhury ST, Bhattacharyya A, Chatterjee M, et al. Author Correction: a multicentric evaluation of dipstick test for serodiagnosis of visceral leishmaniasis in India, Nepal, Sri Lanka, Brazil, Ethiopia and Spain. Sci Rep. 2021;11(1):3967. 33574485 10.1038/s41598-021-83332-8 PMC7878872

[31] Mandrekar JN. Receiver operating characteristic curve in diagnostic test assessment. J Thorac Oncol. 2010;5(9):1315–6. 20736804 10.1097/JTO.0b013e3181ec173d

[32] Kassa M, Abdellati S, Cnops L, Bremer Hinckel BC, Yeshanew A, Hailemichael W, et al. Diagnostic accuracy of direct agglutination test, rK39 ELISA and six rapid diagnostic tests among visceral leishmaniasis patients with and without HIV coinfection in Ethiopia. PLoS Negl Trop Dis. 2020;14(12): e0008963. 33382690 10.1371/journal.pntd.0008963 PMC7774845

[33] Ghosh P, Hasnain MG, Ghosh D, Hossain F, Baker J, Boelaert M, et al. A comparative evaluation of the performance of commercially available rapid immunochromatographic tests for the diagnosis of visceral leishmaniasis in Bangladesh. Parasit Vectors. 2015;8:331. 26077956 10.1186/s13071-015-0935-x PMC4474327

[34] Ejazi SA, Ghosh S, Bhattacharyya A, Kamran M, Das S, Bhowmick S, et al. Investigation of the antigenicity and protective efficacy of *Leishmania* promastigote membrane antigens in search of potential diagnostic and vaccine candidates against visceral leishmaniasis. Parasit Vectors. 2020;13(1):272. 32473634 10.1186/s13071-020-04138-7 PMC7260476

[35] Rezvan H, Moafi M. An overview on *Leishmania* vaccines: a narrative review article. Vet Res Forum. 2015;6(1):1–7. 25992245 PMC4405679

[36] Fernandez L, Carrillo E, Sanchez-Sampedro L, Sanchez C, Ibarra-Meneses AV, Jimenez MA, et al. Antigenicity of *Leishmania*-activated C-kinase antigen (LACK) in Human peripheral blood mononuclear cells, and protective effect of prime-boost vaccination with pCI-neo-LACK plus attenuated LACK-expressing vaccinia viruses in hamsters. Front Immunol. 2018;9:843. 29740446 10.3389/fimmu.2018.00843 PMC5924775

[37] Marlais T, Bhattacharyya T, Pearson C, Gardner BL, Marhoon S, Airs S, et al. Isolation and characterisation of *Leishmania**donovani* protein antigens from urine of visceral leishmaniasis patients. PLoS ONE. 2020;15(9): e0238840. 32925980 10.1371/journal.pone.0238840 PMC7489519

[38] Ghosh P, Bhaskar KR, Hossain F, Khan MA, Vallur AC, Duthie MS, et al. Evaluation of diagnostic performance of rK28 ELISA using urine for diagnosis of visceral leishmaniasis. Parasit Vectors. 2016;9(1):383. 27377266 10.1186/s13071-016-1667-2 PMC4932727

[39] Mahdavi R, Shams-Eldin H, Witt S, Latz A, Heinz D, Fresco-Taboada A, et al. Development of a novel enzyme-linked immunosorbent assay and lateral flow test system for improved serodiagnosis of visceral leishmaniasis in different areas of endemicity. Microbiol Spectr. 2023;11(3): e0433822. 37074181 10.1128/spectrum.04338-22 PMC10269724

[40] Hosseini Farash BR, Mohebali M, Kazemi B, Fata A, Hajjaran H, Akhoundi B, et al. Validation of a mixture of rK26 and rK39 antigens from Iranian strain of *Leishmania**infantum* to detect anti-*Leishmania* antibodies in human and reservoir hosts. Sci Rep. 2022;12(1):10426. 35729270 10.1038/s41598-022-14490-6 PMC9213479

[41] Bezuneh A, Mukhtar M, Abdoun A, Teferi T, Takele Y, Diro E, et al. Comparison of point-of-care tests for the rapid diagnosis of visceral leishmaniasis in East African patients. Am J Trop Med Hyg. 2014;91(6):1109–15. 25311696 10.4269/ajtmh.13-0759 PMC4257631

[42] Kuhne V, Rezaei Z, Pitzinger P, Buscher P. Systematic review on antigens for serodiagnosis of visceral leishmaniasis, with a focus on East Africa. PLoS Negl Trop Dis. 2019;13(8): e0007658. 31415564 10.1371/journal.pntd.0007658 PMC6711545

[43] Cunningham J, Hasker E, Das P, El Safi S, Goto H, Mondal D, et al. A global comparative evaluation of commercial immunochromatographic rapid diagnostic tests for visceral leishmaniasis. Clin Infect Dis. 2012;55(10):1312–9. 22942208 10.1093/cid/cis716 PMC3478143

[44] Fujimori M, Valencia-Portillo RT, Lindoso JAL, Celeste BJ, de Almeida RP, Costa CHN, et al. Recombinant protein KR95 as an alternative for serological diagnosis of human visceral leishmaniasis in the Americas. PLoS ONE. 2023;18(3): e0282483. 36862710 10.1371/journal.pone.0282483 PMC9980733

[45] Singh D, Pandey K, Das VN, Das S, Verma N, Ranjan A, et al. Evaluation of rK-39 strip test using urine for diagnosis of visceral leishmaniasis in an endemic region of India. Am J Trop Med Hyg. 2013;88(2):222–6. 23149580 10.4269/ajtmh.2012.12-0489 PMC3583308

[46] Soares AGR, Landim JS, Franca NG, de Alencar Filho EB, Carmo RF. Differential diagnosis of visceral leishmaniasis in children: a five-year retrospective study at a pediatric referral hospital. BMC Pediatr. 2014;24(1):726. 10.1186/s12887-024-05160-9 PMC11555960 39533219

[47] Diro E, Techane Y, Tefera T, Assefa Y, Kebede T, Genetu A, et al. Field evaluation of FD-DAT, rK39 dipstick and KATEX (urine latex agglutination) for diagnosis of visceral leishmaniasis in northwest Ethiopia. Trans R Soc Trop Med Hyg. 2007;101(9):908–14. 17624385 10.1016/j.trstmh.2007.05.002

[48] Gidwani K, Picado A, Ostyn B, Singh SP, Kumar R, Khanal B, et al. Persistence of *Leishmania donovani* antibodies in past visceral leishmaniasis cases in India. Clin Vaccine Immunol. 2011;18(2):346–8. 21159922 10.1128/CVI.00473-10 PMC3067357

[49] Mollett G, Bremer Hinckel BC, Bhattacharyya T, Marlais T, Singh OP, Mertens P, et al. Detection of immunoglobulin G1 against rK39 improves monitoring of treatment outcomes in visceral leishmaniasis. Clin Infect Dis. 2019;69(7):1130–5. 30541022 10.1093/cid/ciy1062 PMC6743847

[50] Bangert M, Molyneux DH, Lindsay SW, Fitzpatrick C, Engels D. The cross-cutting contribution of the end of neglected tropical diseases to the sustainable development goals. Infect Dis Poverty. 2017;6(1):73. 28372566 10.1186/s40249-017-0288-0 PMC5379574

